# Clinical features and outcomes of congenital chylothorax: a single tertiary medical center experience in China

**DOI:** 10.1186/s13019-022-02009-z

**Published:** 2022-10-27

**Authors:** Beibei Wang, Yun Feng, Yan Guo, Qing Kan, Yunsu Zou, Yue Wu, Mingming Zheng, Rui Cheng

**Affiliations:** 1 Department of Neonatology, Children’s Hospital of Nanjing Medical University, 72 Guangzhou Road, Nanjing, 210008 Jiangsu China; 2 Department of Obstetrics and Gynecology, The Affiliated Drum and Tower Hospital of Medical School of Nanjing University, Nanjing, China

**Keywords:** Congenital chylothorax, Hydrops fetalis, Prenatal intervention, Neonate

## Abstract

**Objective:**

Congenital chylothorax (CC) is an uncommon congenital disease. The objective of this study was to analyze the clinical features, treatment, and outcome of infants with CC in a Chinese tertiary medical center.

**Methods:**

CC was defined as a non-traumatic pleural effusion with ≥ 80% lymphocytes detected before birth or within 28 days after birth. Clinical data were collected in CC infants discharged from June 2017 to March 2021.

**Results:**

A total of 24 CC infants were discharged during the study period, accounting for 67% of congenital pleural effusions. The median gestational age at birth was 36^+4^ weeks (range 29^+5^–41 weeks) and the birth weight was 3025 g (range 1850–4250 g). Twenty-one infants were diagnosed antenatally. The median gestational age at the time of diagnosis was 30^+3^ weeks (range 24–36^+6^ weeks). Nine infants presented with hydrops fetalis; 18 were bilateral. Prenatal interventions were performed in 13 fetuses. Nine infants (38%) had birth asphyxia. Compared with the infants without hydrops fetalis, the infants with CC and hydrops fetalis had lower Apgar scores at 1 and 5 min (*P* < 0.05) and a lower gestational age at birth (*P* < 0.05). Postnatally, 17 infants required continuous pleural drainage for 10 days (range 2–30 days). Analysis of the pleural effusion showed a higher cell count, lymphocyte fraction, and protein content after enteral feeding (*P* < 0.05). Fifteen infants required mechanical ventilation; 9 did not require any respiratory support. Ten infants received a delayed feeding strategy and 17 received a medium-chain triglyceride (MCT) formula. Only 1 infant received octreotide therapy. Twenty-one infants survived and 3 died. The main cause of death was pulmonary dysplasia. The duration of hospital stay in survivors was 21.5 days (range 10–43) days. For infants with CC and hydrops fetalis, prenatal therapy shortened the duration of pleural drainage and the length of hospital stay (*P* < 0.05).

**Conclusion:**

CC is the most common cause of congenital pleural effusions. The poor prognosis is mainly associated with prematurity, hydrops fetalis, and pulmonary dysplasia. Prenatal intervention may improve the outcome of infants with hydrops fetalis.

## Introduction

Congenital chylothorax (CC) refers to the accumulation of pleural lymph fluid, which can be caused by direct leakage of lymph from the thoracic duct, or by overproduction or by obstruction of drainage [1]. It is estimated that the incidence of CC is 1/10000–24,000 [2]. CC is the most common cause of pleural effusion in the neonatal period, with an overall survival rate of 30–70% [2]. Severe cases of CC require respiratory support with a prolonged course and significant mortality.

CC can be detected in the fetal or neonatal period. In the fetal stage, the accumulation of pleural effusion increases intrathoracic pressure, resulting in decreased fetal swallowing of amniotic fluid, which in turn leads to polyhydramnios and preterm labor. Fetal chylothorax may also increase the risk of death and complications from lymphatic accumulation in the pleural space, which compromises lung development, and pulmonary and cardiovascular function, and leads to complications caused by loss of draining lymphatic content. Indeed, prenatal intervention might improve survival of premature infants with CC [1, 3]. The postnatal treatment of CC is usually based on supportive therapy, which includes pleural effusion drainage, mechanical ventilation, replacement of albumin and globulin loss, prevention of infection, and dietary adjustment. Although there have been many summaries on the treatment of CC in recent years, the management of CC remains controversial.

In this study, we analyzed the demographic data, clinical features, treatment, and outcomes of CC infants over the past 4 years in the Children's Hospital of Nanjing Medical University, a tertiary children's hospital in Jiangsu province, to determine the clinical characteristics of CC.

## Methods

This was a retrospective study approved by the Medical Ethics Committee of the Children’s Hospital of Nanjing Medical University. All neonates (≤ 28 days of age) discharged from the Neonatal Medical Center at the Children’s Hospital of Nanjing Medical University from June 2017 to March 2021 were identified from the medical database. Infants diagnosed with a pleural effusion or chylothorax were retrospectively reviewed. The diagnosis of chylothorax was based on an analysis of the pleural effusion, in which the white blood cell count was > 1000/μL and the lymphocyte fraction ≥ 80% [2, 4, 5]. CC was defined as a non-traumatic pleural effusion with ≥ 80% lymphocytes detected before birth or within 28 days after birth. Hydrops fetalis was defined as an abnormal fluid accumulation within at least two extravascular fetal compartments as a pleural effusion, ascites, pericardial effusion, and generalized skin thickening [6].

Demographic data, perinatal management, and clinical outcomes were collected and analyzed. Perinatal management included prenatal diagnosis, prenatal intervention, intrapartum management, and postnatal therapy. Prenatal intervention included thoracocentesis (TC), thoracoamniotic shunting (TAS), and pleurodesis. Intrapartum management refers to neonatal management provided upon delivery before the umbilical cord is cut and the fetal-placental unit is still functioning. Postnatal therapy included respiratory support, effusion drainage, nutritional support, and surgical intervention.

Data were processed using SPSS software (version 23.0). Non-normal distributed data are reported as a median (range). Count data are expressed by the number of cases and the percentage (%). The t-test was used for comparing continuous variables and the chi-square test or Fisher’s exact test was used for comparing categorical variables. All statistical tests were two-tailed and a *p-value* < 0.05 was considered statistically significant.

## Results

A total of 52 infants with pleural effusions were collected during the study period, including 36 with congenital pleural effusions and 16 with acquired pleural effusions, of which 24 were identified as CC and 5 had acquired chylothorax. CC accounted for 67% of congenital pleural effusions and 83% of all chylothorax. Among infants with CC, 13 were males and 11 were females. The median gestational age at birth was 36^+4^ weeks (range 29^+5^–41 weeks) and the median birth weight was 3025 g (range 1850–4250 g). There were 11 full-term and 13 premature infants (5 < 34 weeks gestation and 1 < 32 weeks gestation). Eight infants were delivered vaginally and 16 by caesarean section. Nine infants (38%) had birth asphyxia, including 8 with mild asphyxia and 1 with severe asphyxia. The median Apgar score was 8 (range 3–10) at 1 min and 9 (range 6–10) at 5 min.

Twenty-one infants (88%) were diagnosed antenatally at a median gestational age at the time of diagnosis of 30^+3^ weeks (range 24–36^+6^ weeks). Nine infants (38%) presented with hydrops fetalis. Effusions were bilateral in 18 infants, unilateral in 6 infants (2 on the left and 4 on the right). Prenatal intervention was performed in 13 infants (54%). All 13 infants received TC as an initial procedure and 8 received TAS due to re-accumulation of fluid. The median interval between the first intervention to delivery was 14 days (range 3–56 days). Thirteen infants, including 2 non-survivors, had chromosome examinations antenatally that revealed no abnormalities.

We compared the clinical features of infants with or without hydrops, as shown in Table 1. Compared with infants without fetal hydrops, infants with hydrops had lower Apgar scores at 1 and 5 min (*P* < 0.05) and gestational age at birth (*P* < 0.05). No differences existed with respect to other ante-, intra-, and post-partum factors. The in-hospital survival rates in infants with and without hydrops were 78% and 93%, respectively.

**Table 1:** Perinatal characteristics of CC infants with or without hydrops fetalis

Hydrops fetalis	With (n = 9)	Without (n = 15)	*p* value
Male	5	8	1.000
Gestational age at birth(weeks)	34^+4^ (29^+5^–37^+2^)	37^+2^ (33^+3^–41)	0.007*
Birth weight (g)	3000 (1900–3770)	3112 (1850–4250)	0.842
Cesarean section	5	9	1.000
Gestational age of diagnosis (weeks)	30 (24 + 1–36 + 6)	31 (24–36 + 6)	0.811
Prenatal therapy	6	7	0.423
Bilateral chylothorax	8	10	0.351
1-min Apgar score	6 (3–9)	9 (6–10)	0.001*
5-min Apgar score	8 (6–9)	10 (6–10)	0.027*
Duration of mechanical ventilation (days)	6 (2–16)	5 (1–9)	0.231
Duration of pleural drainage (days)	13 (2–27)	10 (7–30)	0.959
Length of hospital stay (days)	23 (2–43)	19 (1–40)	0.486
Survival to discharge	7	14	0.533

Fourteen infants (54%) received endotracheal intubation and 2 (8%) received TC immediately after birth in the delivery room. After birth, 6 infants only received TC and 17 needed continuous pleural drainage with a median duration of 10 days (range 2–30 days). Analysis of pleural effusions before and after enteral feeding was performed in 16 and 13 infants, respectively. Pleural fluid was clear in unfed infants, but appeared creamy in fed patients. The characteristics of the pleural effusions are described in Table 2. After feeding, the cell count, lymphocyte fraction, and protein content of the pleural effusions were significantly higher than unfed infants (*P* < 0.05).

**Table 2:** Characteristics of pleural fluid in infants with CC

Enteral feeding	With	Without	*p* value
Cell count (/μl)	2716 (609–5577)	16,881 (1933–32,788)	< 0.0001*
Lymphocyte fraction (%)	96.35 (72–100)	98.4 (90.7–99.8)	0.026*
Protein (g/L)	19.85 (9.2–29.5)	35.3 (21.5–45.1)	< 0.0001*
Lactate dehydrogenase (mmol/L)	215 (115–634)	238 (158–560)	0.640
Glucose (mmol/L)	4.3 (2.1–6.91)	4.63 (2.77–5.48)	0.893
Chloride (mmol/L)	107.8 (101–113)	104.1 (100.8–113)	0.988

Fifteen infants (63%) required mechanical ventilation (MV), including 4 using a high-frequency ventilator and 4 using inhaled nitric oxide. The maximal mean airway pressure was 11.3 cmH_2_O (range 10–19cmH_2_O) and the median duration of MV was 7 days (range 1–16 days). Nine infants did not require any respiratory support. Six infants (25%) developed pneumothorax and 3 occurred within 24 h after birth. Surfactant instillation was provided to 7 infants (29%). All the infants with a gestational age ≤ 34 weeks at birth were treated with MV and surfactant instillation.

Ten infants (42%) received a delayed feeding strategy. The median enteral nutrition start time was 6 days (range 5–10 days). Seventeen infants (71%) received a medium-chain triglyceride (MCT) diet at some time during the course of treatment. The patients were converted to normal formula or breastfeeding with a median time of 4 months (range 1.5–12 months) after ultrasound showing that the pleural effusion did not recur. Recurrence of pleural effusion appeared in 2 infants during conversion.

Thirteen infants (54%) received albumin replacement therapy, 9 (38%) received immunoglobulins, 13 (54%) received fresh frozen plasma, and 3 (10%) received a blood transfusion. Only 1 infant received octreotide therapy (1–3 μg/kg/h) for 3 weeks. No potential adverse effects were noted.

Twenty-one infants (88%) survived and 3 (12%) died. Two infants died of pulmonary dysplasia. Another infant was difficult to withdraw from the ventilator and the guardian requested to discontinue treatment; the infant died 2 days later. The mean duration of hospital stay in survivors was 21.5 days (range 10–43 days).

Because hydrops fetalis is a severe form of CC, we compared the clinical parameters in CC infants with hydrops fetalis who did or did not receive prenatal therapy (Table 3). Indeed, prenatal therapy shortened the duration of pleural drainage and length of hospital stay (*P* < 0.05).

**Table 3:** Demographic data and clinical variables in CC infants with hydrops fetalis with or without prenatal therapy

Prenatal therapy	With (n = 6)	Without (n = 3)	*p* value
Male	3	2	1.000
Gestational age at birth (weeks)	33^+5^ (29^+5^–37^+2^)	35^+1^ (34^+1^–36^+5^)	0.391
Birth weight (g)	2830 (1900–3410)	3320 (3000–3770)	0.142
Cesarean section	2	3	0.167
Bilateral chylothorax	5	2	1.000
1-min Apgar score	6.5 (3–9)	6 (5–6)	0.699
5-min Apgar score	8.5 (6–9)	8 (7–9)	1.000
Ventilator use	5	3	1.000
MV duration (days)	6 (0–16)	7 (5–11)	0.718
Duration of pleural drainage (days)	4 (0–16)	25 (16–27)	0.005*
Maximal MAP(cmH2O)	12.5 (9.3–19)	14.8 (8.8–18)	0.876
OI onset	17.35 (7–32.8)	31.67 (13.34–50)	0.403
iNO	1	1	1.000
Surfactant instillation	4	1	0.524
Length of hospital stay (days)	16 (2–33)	42 (30–43)	0.018*
Mortality	1	0	1.000

iNO, inhaled nitric oxide; MV, mechanical ventilation; OI, oxygenation index; MAP, mean airway pressure

## Discussion

CC can occur alone or in combination with genetic syndromes and other anomalies [3]. The most common genetic syndromes were Down, Noonan, and Turner syndromes, and the most common associated anomalies were pulmonary lymphangiectasia and pulmonary hypoplasia [3, 7]. A prospective epidemiological survey in Germany showed that 24% of CC infants had syndromal anomalies, the most common being Noonan syndrome [8]. No combination of proven or suspected genetic syndromes were identified in our cases, which may be related to the improvement in prenatal diagnostic techniques in our geographic region in recent years. CC can occur at any stage of pregnancy, most often in the third trimester [3, 9]. Once a fetal pleural effusion is detected, ultrasonic examination of fetal anatomic structures and fetal echocardiography should be performed to exclude other deformities. Prenatal evaluations also include fetal karyotype and/or chromosomal microarray analysis, maternal blood count and grouping with antibody status, and virology screening, including TORCH and parvovirus B19 [10, 11].

Prematurity, hydrops fetalis, and pulmonary dysplasia are related to a poor prognosis in infants with CC, and the most common reason for death is pulmonary dysplasia [12]. In our study, the two early deaths among CC infants were both attributed to pulmonary dysplasia, which was inconsistent with the literature. Recently, attempts have been made to develop prenatal prediction of pulmonary dysplasia by prenatal ultrasound and fetal magnetic resonance, but clinical applications are limited [13]. It has been reported that survival is high in infants delivered after 32 weeks gestation [1]. The survival rate in infants with and without hydrops fetalis reported in our study was 78% and 88%, respectively, which was similar to that reported by Tai et al. [9], but higher than previous reports of 30–70% [2]. In addition to gestational age, prenatal intervention is an important factor affecting the prognosis of infants with CC.

The goal of prenatal interventions is to reduce the mass effects of pleural effusion, and to reduce the risk of pulmonary dysplasia. Prenatal interventions include ultrasound-guided fetal TC, TAS, and pleurodesis. Although there are no randomized controlled trials, retrospective data support that prenatal intervention improves perinatal survival [1, 3, 14], with TAS superior to TC [3, 11]. In our study, 13 infants were treated with TC and 8 received TAS due to fluid re-accumulation. Due to the small number of cases and differences in fetal status, our study was not able to compare the efficacy of different prenatal interventions. Intrapartum extrauterine therapy (EXIT) has also been introduced in infants with CC. EXIT refers to respiratory management prior to severing the umbilical cord, including endotracheal intubation and thorax puncture, to leave sufficient space for lung expansion before the infant starts effective spontaneous breathing [9, 15]; however, an early pneumothorax often occurs in infants who received intrapartum TC alone. An early pneumothorax is associated with a poor outcome in infants with CC [16]. In our study, two infants received EXIT with TC alone, one of which was complicated with early-onset pneumothorax that was not life-threatening.

Postnatal management of CC includes respiratory support, pleural drainage, nutritional management, and surgery. The choice of treatment regimen depends on the response to treatment by assessing the daily amount of thoracic drainage and the degree of interference with pulmonary function [2].

The majority of CC infants need ventilatory support to treat CC-related respiratory failure in the neonatal period. Both conventional and high-frequency ventilation have been used; however, it is difficult to compare the effectiveness of the various ventilation modes in the treatment of CC. High-frequency oscillatory ventilation (HFOV) has been suggested in severe cases because HFOV improves lung opening and volume maintenance, possibly shortening lymph flow over time [17]. In our study, a majority (63%) of infants required mechanical ventilation. Four severely-affected infants received HFOV during the course of treatment, of whom two survived and one developed with bronchopulmonary dysplasia (BPD), which may be attributed to pulmonary dysplasia.

The purpose of nutritional management is to decrease lymphatic flow. Treatment options include a delayed feeding strategy and MCT formula. Long-chain fatty acids are transported in the form of chylomicrons by the lymphatic system. High fat intake increases lymphatic flow significantly. An MCT formula contains little-to-no fat, thus by passing intestinal lymphatic processing and are absorbed directly into the portal venous system [18]. An MCT formula is gradually introduced by the end of the first week after birth [19]. An MCT formula is often continued for a period of 6 months until a pleural effusion does not recur.

Octreotide is a somatostatin analogue that is also commonly used to reduce lymphatic flow. Octreotide is introduced when pleural drainage and diet modification are ineffective. The recommended therapeutic dose and response of octreotide reported in the literature are mixed, often with intravenous infusion at a starting dose of 1 μg/kg/h and gradually increasing to 10 μg/kg/h, according to the therapeutic response [19, 20]. Side effects and complications include hyperglycemia, abdominal distension, bloody stools, and pulmonary hypertension [20]. It has been suggested that high doses of octreotide can be used to treat refractory chylothorax, and the dose of octreotide can be safely titrated to a maximum of 20ug/kg/h with no significant side effects [21, 22].

Tamaoka et al. [23] successfully eliminated effusion by using midodrine, an oral alpha-1 adrenoreceptor agonist, in a patient with refractory chylothorax. Tomobe et al. [24] used combination therapy with etilefrine and pleurodesis for refractory CC. The emergence of these new drugs provides more options for the clinical treatment of refractory chylothorax, but most are reports of individual cases. The efficacy and safety of these drugs need to be further evaluated.

Surgical intervention, including chemical pleurodesis, is the final treatment to cure CC. Most CC infants achieve complete remission 3–4 weeks after birth. Surgical intervention should be considered around 6 weeks when conservative treatment fails [7]. OK-432 is an inactivated preparation of human-derived *Streptococcus pyogenes* with immunological activity and is often used to induce aseptic inflammation of the pleura [7]. In addition to extrauterine treatment, OK-432 pleurodes is also successfully used for fetal pleural effusions in utero [25].

## Conclusion

Our study summarized the experience of our center in the treatment of CC over the past four years. There was a higher survival rate, which may be related to improvements in prenatal diagnosis and interventions in recent years. There were some limitations in this study. First, this was a retrospective study and the data collection was incomplete. Second, we did not follow the assessment of neurologic development. Third, there were no cases of pleurodesis in our study and octreotide therapy was conducted in only one case, thus the relevant clinical experience was insufficient. We need additional prospective population-based epidemiologic, treatment and outcome data on CC.

## Data Availability

All data generated or analyzed during this study are included in this published article.

## Abbreviations

CC: Congenital chylothorax
TC: Thoracocentesis
TAS: Thoracoamniotic shunting
MV: Mechanical ventilation
MCT: Medium-chain triglyceride
EXIT: Intrapartum extrauterine therapy
HFOV: High-frequency oscillatory ventilation;
BPD: Bronchopulmonary dysplasia
LFBM: Breast milk with low fat content
